# Incidence and outcomes of pregnancy-associated cancer in Australia, 1994–2008: a population-based linkage study

**DOI:** 10.1111/j.1471-0528.2012.03475.x

**Published:** 2012-09-05

**Authors:** YY Lee, CL Roberts, T Dobbins, E Stavrou, K Black, J Morris, J Young

**Affiliations:** 1 Clinical and Population Perinatal Health Research, Kolling Institute of Medical Research, University of SydneySydney, NSW; 2 Cancer Epidemiology and Services Research Group, Sydney School of Public Health, University of SydneySydney, NSW; 3 Adult Cancer Program, Lowy Cancer Research Centre, University of New South WalesSydney, NSW; 4 Department of Obstetrics and Gynaecology, University of SydneySydney, NSW, Australia

**Keywords:** Cancer, cohort study, incidence, pregnancy, record linkage

## Abstract

**Objective:**

To determine trends in pregnancy-associated cancer and associations between maternal cancer and pregnancy outcomes.

**Design:**

Population-based cohort study.

**Setting:**

New South Wales, Australia, 1994–2008.

**Population:**

A total of 781 907 women and their 1 309 501 maternities.

**Methods:**

Cancer and maternal information were obtained from linked cancer registry, birth and hospital records for the entire population. Generalised estimating equations with a logit link were used to examine associations between cancer risk factors and pregnancy outcomes.

**Main outcome measures:**

Incidence of pregnancy-associated cancer (diagnosis during pregnancy or within 12 months of delivery), maternal morbidities, preterm birth, and small- and large-for-gestational-age (LGA).

**Results:**

A total of 1798 new cancer diagnoses were identified, including 499 during pregnancy and 1299 postpartum. From 1994 to 2007, the crude incidence rate of pregnancy-associated cancer increased from 112.3 to 191.5 per 100 000 maternities (*P* < 0.001), and only 14% of the increase was explained by increasing maternal age. Cancer diagnosis was more common than expected in women aged 15–44 years (observed-to-expected ratio 1.49; 95% CI 1.42–1.56). Cancers were predominantly melanoma (33.3%) and breast cancer (21.0%). Women with cancer diagnosed during pregnancy had high rates of labour induction (28.5%), caesarean section (40.0%) and planned preterm birth (19.7%). Novel findings included a cancer association with multiple pregnancies (adjusted odds ratio 1.52, 95% CI 1.13–2.05) and LGA (aOR 1.47, 95% CI 1.14–1.89).

**Conclusions:**

Pregnancy-associated cancers have increased, and this increase is only partially explained by increasing maternal age. Pregnancy increases women’s interaction with health services and the possibility for diagnosis, but may also influence tumour growth.

## Introduction

Cancer is the second leading cause of death in women during their reproductive years. The incidence is generally reported to be one for every 1000 maternities.^1^–^5^ Despite its rarity, the trend of women postponing childbearing to older age has raised concern that the incidence of cancer in pregnancy is likely to increase.^2^,^3^,^5^–^8^

Pregnancy-associated cancer refers to instances in which the initial diagnosis of cancer is made during pregnancy or within 12 months of delivery.^1^,^6^,^9^–^11^ The rationale for including cancers that are diagnosed after pregnancy are: women and physicians may incorrectly attribute cancer-related symptoms to the physiologic changes of pregnancy; there is reluctance to perform radiographs or invasive procedures during pregnancy, leading to delayed diagnosis; and less aggressive tumours are more likely to remain undetected until after delivery.^1^

To date, estimates of the incidence of pregnancy-associated cancer have been imprecise, for example, malignant melanoma is reported to affect between one in 1000 and one in 10 000 maternities, breast cancer is reported to affect between one in 3000 and one in 10 000 maternities, and ovarian cancer is reported to affect between one in 10 000 and one in 100 000 maternities.^2^,^4^,^8^ A population-based Californian study identified pregnancy-associated cancers with hospital data, and was then repeated with linked hospital and cancer registry data. The study showed that cancer ascertainment from the population-based statutory cancer register produced more refined and reliable incidence estimates.^1^,^9^ However, the majority of published incidence estimates of pregnancy-associated cancer have come from case reports or small case studies, and have relied on data collected before the year 2000. Consequently, questions regarding the population incidence remain. In addition, few studies have examined the associated pregnancy outcomes; to date, the majority have focused on one cancer type, and their sample sizes have been small.^7^,^10^–^13^

Accurate reporting of the incidence of pregnancy-associated cancer and the associated pregnancy outcomes is important for informing treatment and counselling for women. Our study had two aims: first, to determine recent trends in the incidence of pregnancy-associated cancer and the impact of increasing maternal age; and second, to compare risk factors and pregnancy outcomes for women with and without pregnancy-associated cancer.

## Methods

The study population comprised 781 907 women who gave birth in New South Wales (NSW) in the period 1994–2008, which corresponded with 1 309 501 maternities and 1 329 306 infants. With a resident population of nearly 7 million people, NSW is the most populous state of Australia. Approximately one-third of all Australian births occur in NSW.

### Data sources

Data were obtained from three linked NSW population databases: the Perinatal Data Collection (PDC); Central Cancer Registry (CCR); and Admitted Patient Data Collection (APDC). Record linkage was carried out by the NSW Centre for Health Record Linkage. As Australia does not have a unique registration number for citizens, the separate data sets were linked using probabilistic linkage methods.^14^,^15^ This involves a process of blocking and matching combinations of selected variables such as name, date of birth, address and hospital, and assigning a probability weight to the match. The validity of the probabilistic record linkage is extremely high, with <3 in 1000 false-positive links, and <5 in 1000 missed links.^14^ The researchers were provided anonymised data. Ethics approval for the study was obtained from the NSW Population and Health Services Research Ethics Committee.

The PDC is a legislated population-based surveillance system that includes births of at least 20 weeks of gestation or with birthweights of at least 400 g. Information is recorded by either the midwife or medical practitioner providing maternity care, and includes demographic, medical and obstetric information on the mother, as well as details of labour, delivery and condition of the neonate.

The CCR is a statutory case-based registry that includes demographic, cancer diagnosis and mortality information for every new cancer diagnosed in NSW since 1972, with the exception of non-melanoma skin cancers. Information is recorded by the treating clinicians in public and private hospitals, departments of radiation oncology and pathology laboratories. Diagnosis, topography and morphology for each cancer notification are coded according to the third edition of the International Classification of Diseases for Oncology.^16^ Over 90% of cancers are verified by pathology and are confirmed as the primary diagnosis.^17^ To preserve the privacy and confidentiality of the individuals, only the month and year of diagnosis are available in the registry.

The APDC is a census of all hospitalisations, and includes summary discharge information for every inpatient admission to NSW public and private hospitals. Diagnoses and procedures for each admission are coded according to the tenth revision of the International Classification of Disease, Australian Modification and the Australian Classification of Health Interventions.^18^

The PDC birth records from 1994 to 2008 were linked to the maternal cancer notifications to identify a cohort of women with newly diagnosed cancer during pregnancy or within 12 months of delivery.^1^,^6^,^9^–^11^ Linkage to the maternal birth-related hospital records was only available from 2000 to 2008, but allowed the assessment of adverse pregnancy outcomes and antenatal hospitalisations for that period.

### Study factors

Cancers were categorised into 13 clinical groupings based on treatment categories.^17^ According to the international coding guidelines,^19^ stage at diagnosis was defined as the highest degree of spread that occurs within 4 months from the date of cancer diagnosis. Pregnancy-associated cancers were stratified depending on the time of initial diagnosis as follows: ‘Pregnancy’ if the diagnosis was made between conception and delivery or ‘Postpartum’ if the diagnosis was made within 12 months of delivery.

Sociodemographic information included maternal age, country of birth and, based on postcode of residence, socio-economic status (Index of Relative Socio-economic Disadvantage)^20^ and rural/remoteness (Accessibility/Remoteness Index for Australia).^21^ Pregnancy information included plurality and parity. (Note that for the purpose of this study, nulliparity refers to cancers in the first pregnancy and in the 12 months postpartum; Table 2). Maternal factors included hypertensive disorders (chronic or gestational hypertension and pre-eclampsia or eclampsia) and diabetes (pre-existing or gestational), as well as the use of assisted reproductive technologies. All the above information was obtained from the birth records except for the use of assisted reproductive technologies, which was obtained from the linked maternal birth-related hospital records, and was only available for births occurring from 2001 onwards.

**Table 2:** Maternal characteristics of women with cancer diagnosed during pregnancy or postpartum compared to pregnant women without cancer, among 1 309 501 maternities, New South Wales, 1994–2008

Maternal characteristic	Pregnancy *N* = 495 *n* (%)	Postpartum *N* = 1290 *n* (%)	All *N* = 1785 *n* (%)	No cancer *N* = 1 307 716 *n* (%)	Crude OR (95% CI)	Adjusted OR (95% CI)
**Maternal age**						
<30 years	147 (29.7)	362 (28.1)	509 (28.5)	654 593 (50.1)	Reference	Reference
30–34 years	172 (34.7)	505 (39.1)	677 (37.9)	413 643 (31.6)	2.10 (1.87, 2.36)	2.07 (1.84, 2.33)
35–39 years	137 (27.7)	332 (25.7)	469 (26.3)	200 242 (15.3)	3.01 (2.65, 3.42)	2.98 (2.61, 3.40)
≥40 years	39 (7.9)	91 (7.1)	130 (7.3)	38 615 (3.0)	4.34 (3.58, 5.26)	4.34 (3.57, 5.29)
Missing	0 (0.0)	0 (0.0)	0 (0.0)	623 (0.0)	–	–
**Australian born**						
Yes	384 (77.6)	967 (75.0)	1351 (75.7)	945 444 (72.3)	1.19 (1.07, 1.33)	1.35 (1.21, 1.51)
No	110 (22.2)	321 (24.9)	431 (24.1)	359 634 (27.5)	Reference	Reference
Missing	1 (0.2)	2 (0.2)	3 (0.2)	2638 (0.2)	–	–
**Socio-economic status (IRSD)**						
Lowest, 2nd–4th quintiles	362 (73.1)	1001 (77.6)	1363 (76.4)	1 064 664 (81.4)	Reference	Reference
Highest 5th quintile	133 (26.9)	284 (22.0)	417 (23.4)	230 856 (17.7)	1.41 (1.26, 1.58)	1.14 (1.02, 1.28)
Missing	0 (0.0)	5 (0.4)	5 (0.3)	12 196 (0.9)	–	–
**Remoteness (ARIA+)**						
Urban	444 (89.7)	1156 (89.6)	1600 (89.7)	1 153 954 (88.3)	Reference	Not retained
Rural	51 (10.3)	130 (10.1)	181 (10.1)	150 882 (11.5)	0.87 (0.74, 1.01)	
Missing	0 (0.0)	4 (0.3)	4 (0.2)	2880 (0.2)	–	
**Parity**						
0	288 (58.2)	678 (52.6)*	966 (54.1)	780 754 (59.7)	Reference	Reference
≥1	207 (41.8)	612 (47.4)	819 (45.9)	526 962 (40.3)	1.27 (1.15, 1.39)	1.10 (1.00, 1.21)
**Plurality**						
Singleton	484 (97.8)	1256 (97.4)	1740 (97.5)	1 288 370 (98.5)	Reference	Reference
Multiple pregnancy	11 (2.2)	34 (2.6)	45 (2.5)	19 346 (1.5)	1.72 (1.28, 2.32)	1.52 (1.13, 2.05)
**Diabetes**						
Yes	21 (4.2)	87 (6.7)	108 (6.1)	56 302 (4.3)	Not applicable, timing of onset unknown	
No	474 (95.8)	1203 (93.3)	1677 (93.9)	1 251 414 (95.7)		
**Hypertensive disorders**						
Yes	38 (7.7)	96 (7.4)	134 (7.5)	88 130 (6.7)	Not applicable, timing of onset unknown	
No	457 (92.3)	1194 (92.6)	1651 (92.5)	1 219 586 (93.3)		
**Previous cancer (same or different type)**						
Yes	7 (1.4)	23 (1.8)	30 (1.7)	4454 (0.3)	2.78 (1.43, 5.41)	1.98 (1.00, 4.01)
No	488 (98.6)	1267 (98.2)	1755 (98.3)	1 303 262 (99.7)	Reference	Reference
**Assisted reproductive technology**						
Yes	10 (2.0)	27 (2.1)	37 (2.1)	13 301 (1.0)	1.94 (1.40, 2.70)	Not retained
No	485 (98.0)	1263 (97.9)	1748 (97.9)	1 294 415 (99.0)	Reference	
**Stage of cancer**						
*In situ*	51 (10.3)	119 (9.2)	170 (9.5)	Not applicable		
Localised	230 (46.5)	622 (48.3)	852 (47.8)			
Regionalised	96 (19.4)	256 (19.8)	352 (19.7)			
Distant	21 (4.2)	83 (6.4)	104 (5.8)			
Unknown	97 (19.6)	210 (16.3)	307 (17.2)			

ARIA+, Accessibility Index for Australia; CI, confidence interval; IRSD, Index of Relative Socio-economic Disadvantage; OR, odds ratio.

Adjusted for maternal age, country of birth, socio-economic status, parity, plurality and previous cancer. Two women with two cancers diagnosed during pregnancy. Three women with two cancers and one with three cancers diagnosed during postpartum.

* Postpartum diagnosis after the first birth.

Pregnancy outcomes from the PDC included induction of labour, mode of delivery (spontaneous vaginal birth, instrumental birth and caesarean section) and place of birth. Other pregnancy outcomes and cancer complications included antenatal hospitalisation, obstetric haemorrhage (antepartum or postpartum), thromboembolic events (antepartum pulmonary embolism, puerperal pulmonary embolism, cerebral ischaemia or infarction and puerperal deep vein thrombosis), sepsis (septicaemia, group B streptococcal or gram-negative sepsis) and severe morbidity, which were obtained from diagnosis and procedure codes in the linked pregnancy and postpartum hospital records. Severe maternal morbidity was measured using a validated composite indicator relating to serious adverse maternal health outcomes such as transfusion, pulmonary embolism, hysterectomy and mechanical ventilation.^22^ Antenatal hospitalisation was defined as an admission prior to and without birth, or an admission where a birth occurs with an admission date at least 4 days prior to the date of delivery.^23^ Perinatal outcomes included spontaneous preterm birth (<37 weeks of gestation), planned preterm birth (induction of labour or prelabour caesarean before 37 weeks of gestation), perinatal death and size at birth, obtained from the birth records. Small for gestational age (SGA, <10th percentile)^24^ was of interest as a potential consequence of cancer or cancer treatment. Large for gestational age (LGA, >90th percentile)^24^ is a recognised risk factor for infant and childhood cancers, and was pre-specified as potentially associated with maternal cancer. Only variables that are clearly and accurately reported were included in the analyses.^25^–^28^

### Statistical analyses

The crude incidence rates of pregnancy-associated cancer were calculated by dividing the number of newly diagnosed cancers during pregnancy and postpartum by the number of maternities, in which multifetal maternities were counted once. Each notification in the cancer registry is a primary diagnosis, and more than one cancer in a pregnancy is possible. The crude rates were then standardised to the population in 1994 to obtain the direct age-standardised rates. All rates were expressed per 100 000 maternities.

To assess whether pregnancy increased the risk of cancer or cancer diagnosis, we compared the number of pregnancy-associated cancers with the number expected based on the population incidence for all women aged 15–44 years.^29^ We used indirect standardisation by 5-year age groups for the five most common pregnancy-associated cancers. The observed-to-expected ratios with 95% confidence intervals were estimated assuming a Poisson distribution for the observed frequencies.

Cochran–Armitage trend tests were used to assess for a linear trend in incidence rates of pregnancy-associated cancer, both overall and by maternal age group, for births occurring in 1994–2007. Births occurring in 2008 were excluded because these women did not have 12 months of postpartum data, which would lead to the under-reporting of incidences of cancer for that year. Crude associations between maternal cancer and each outcome were examined via cross-tabulation and chi-square tests. Generalised estimating equations with a logit link and an exchangeable correlation matrix were used to examine both risk factors for maternal cancer and maternal cancer as a risk factor for adverse pregnancy outcomes, while accounting for within-women correlation resulting from multiple pregnancies from the same women in the study period. The latter models were adjusted for maternal age, socio-economic status, plurality, parity, diabetes and hypertensive disorders to estimate the adjusted odds ratios (aORs) with 95% confidence intervals (95% CIs). Preterm birth was further adjusted for previous preterm births. Sensitivity analysis was conducted, excluding women with melanoma of skin diagnosed during pregnancy, with no material change to the magnitude of the adjusted odds ratios (data not shown). Analyses were carried out in SAS 9.2 (SAS Institute, Cary, NC, USA).^30^

## Results

### Incidence of pregnancy-associated cancer

**Figure 1:**
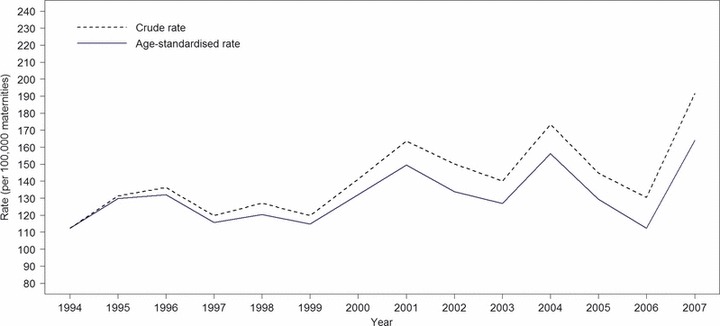
Crude and direct age-standardised incidence rates of pregnancy-associated cancer, NSW, 1994–2007.

Between 1994 and 2008, a total of 1798 pregnancy-associated cancers were identified in 1 309 501 maternities among 781 907 women. This corresponds to an overall crude incidence of 137.3 per 100 000 maternities. Figure 1 shows that from 1994 to 2007 the crude incidence rate of pregnancy-associated cancer increased from 112.3 to 191.5 per 100 000 maternities (*P* < 0.001). During this period maternal age also increased; the percentage of women aged 35 years and over increased from 13.2 to 23.6% (with women aged 40 years and over increasing from 1.9 to 4.0%). The impact of increasing maternal age is demonstrated in the direct age-standardised incidence rates, which fall away from the crude incidence rates as the years advance (Figure 1). By 2007, the age-standardised rate (164.0 per 100 000 maternities) was 14.4% lower than the crude rate (191.5 per 100 000 maternities). Furthermore, the rate per 100 000 maternities increased with increasing maternal age (<30 years, from 70.7 in 1994 to 105.2 in 2007, *P* = 0.05; 30–34 years, from 168.2 in 1994 to 192.8 in 2007, *P* = 0.31; and ≥35 years, from 168.1 in 1994 to 357.0 in 2007, *P* = 0.02).

**Figure 2:**
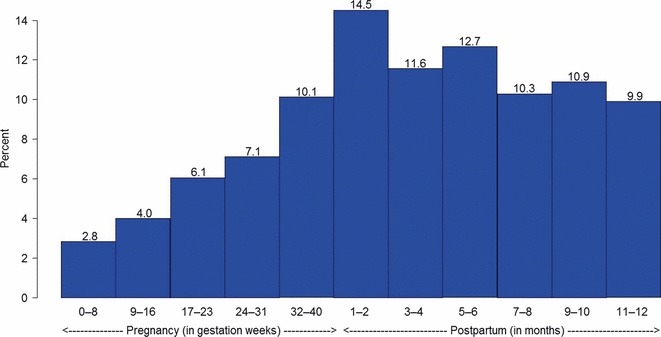
Timing of pregnancy-associated cancer diagnosis, NSW, 1994–2008.

A total of 1767 women had 1785 cancer-affected maternities and 1798 new cancer diagnoses, including 499 during pregnancy and 1299 during the postpartum period. Twenty-nine women had two diagnoses of cancer and one women had three diagnoses of cancer (same or different type), including 12 women with multiple cancers in the same pregnancy and 18 in other pregnancies. Among the study population from 1994 to 2008, there were 18 maternal cancer deaths in the pregnancy group and 24 in the postpartum group. The proportion of cancer (of any type) increased steadily as the duration of pregnancy advanced (Figure 2). The highest proportion of cancer occurred in the 2 months postpartum and declined slowly thereafter. The most common cancers were melanoma of skin (*n* = 599, 45.7 per 100 000 maternities), breast cancer (*n* = 377, 28.8 per 100 000 maternities), thyroid and other endocrine cancers (*n* =228, 17.4 per 100 000 maternities), and gynaecological (*n* = 187, 14.3 per 100 000 maternities) and lymphohaematopoeitic cancers (*n* = 151, 11.5 per 100 000 maternities) (Table 1). These cancers accounted for 85.8% of the observed counts, and most (47.8%) were localised (e.g. melanoma of skin 65.6%), with the exception of colorectal cancer (22.6%). Of the cancers diagnosed during pregnancy, 45% were diagnosed before 20 weeks of gestation.

**Table 1:** The number and crude incidence rate of pregnancy-associated cancer, by the timing of initial diagnosis and clinical group of cancer, NSW, 1994–2008

Clinical group of cancer	Pregnancy		Postpartum *n* (rate)*	All *n* (rate)*
			
	*n* (rate)*	<20 weeks (%)**		
**Melanoma of skin**	198 (15.1)	48.0	401 (30.6)	599 (45.7)
**Breast**	95 (7.3)	33.7	282 (21.5)	377 (28.8)
**Thyroid and other endocrine**	42 (3.2)	61.9	186 (14.2)	228 (17.4)
**Gynecological**	51 (3.9)	37.3	136 (10.4)	187 (14.3)
Cervix	24 (1.8)	50.0	86 (6.6)	110 (8.4)
Ovary	19 (1.5)	21.1	28 (2.1)	47 (3.6)
Other female genital organs	7 (0.5)	42.9	9 (0.7)	16 (1.2)
Uterus and body	1 (0.1)	0.0	7 (0.5)	8 (0.6)
Placenta	0 (0.0)	0.0	6 (0.5)	6 (0.5)
**Lymphohaematopoeitic**	53 (4.0)	47.2	98 (7.5)	151 (11.5)
**Colorectal**	10 (0.8)	20.0	52 (4.0)	62 (4.7)
**Neurological**	12 (0.9)	50.0	33 (2.5)	45 (3.4)
**Bone and other connective tissue**	8 (0.6)	50.0	26 (2.0)	34 (2.6)
**Head and neck**	9 (0.7)	55.6	21 (1.6)	30 (2.3)
**Upper gastrointestinal**	6 (0.5)	16.7	24 (1.8)	30 (2.3)
**Respiratory**	3 (0.2)	0.0	19 (1.5)	22 (1.7)
**Ill-defined and unknown primary sites**	9 (0.7)	55.6	9 (0.7)	18 (1.4)
**Urogenital**	3 (0.2)	66.7	12 (0.9)	15 (1.1)
**Total**	499 (38.1)	44.7	1 299 (99.2)	1 798 (137.3)

* Crude incidence rate per 100 000 maternities.

** Gestational age at diagnosis of <20 weeks among cancers diagnosed during pregnancy.

### Comparison with cancer incidence in the general female population

There were approximately 49% more pregnancy-associated cancers than would have been expected based on the rates in the general female population for women aged 15–44 years from 1994 to 2007. The ratio of observed-to-expected rates for all cancers was 1.49 (95% CI 1.42–1.56). The ratio of observed-to-expected rates for each of the five most common cancers were: melanoma of skin, 2.22 (95% CI 2.05–2.41); followed by thyroid and other endocrine cancers, 1.54 (95% CI 1.35–1.75); lymphohaematopoeitic cancer, 1.36 (95% CI 1.15–1.59); breast cancer, 1.23 (95% CI 1.11–1.36); and gynaecological cancers, 1.20 (95% CI 1.03–1.38).

### Maternal characteristics and risk factors

Although increasing maternal age, being born in Australia, high socio-economic status, multiparity, multiple pregnancy, prior diagnosis of cancer and use of assisted reproductive technology were all crude risk factors for pregnancy-associated cancer, only older maternal age, being born in Australia, high socio-economic status, multiparity, multiple pregnancy and prior diagnosis of cancer were retained as independent risk factors in the multivariable model (Table 2). Maternal age ≥40 years had the highest adjusted odds ratio, but explained only 7.3% of cancers.

### Pregnancy and perinatal outcomes

Women with cancer diagnosed during pregnancy were more likely to deliver at a tertiary hospital, and to have an induced labour and prelabour caesarean sections, including those performed preterm (Table 3). The mean gestational age at delivery was 37.7 weeks (SD = 3.2 weeks) for women with cancer diagnosed during pregnancy, 38.8 weeks (SD = 2.3 weeks) for women with cancer diagnosed postpartum and 39.0 weeks (SD = 2.2 weeks) for women without cancer. Antenatal hospitalisations were more frequent for women with cancer diagnosed during pregnancy: 33.8% had one and 30.7% had two or more admissions, compared with 16.8 and 8.7%, respectively, in women without cancer. Rates of LGA infants were increased among women with cancer during or after pregnancy, but perinatal death rates were similar among women with or without cancer (Table 3). After adjusting for other risk factors, cancer during pregnancy was associated with a significantly increased risk of caesarean section, planned preterm birth and LGA infants (Table 3). A significant LGA association was observed for melanoma of skin (aOR 2.00, 95% CI 1.42–2.83), but not for breast (aOR 0.84, 95% CI 0.44–1.72), gynaecological (aOR 1.59, 95% CI 0.78–3.24), lymphohaematopoeitic (aOR 1.10, 95% CI 0.45–2.68), and thyroid and other endocrine cancers (aOR 1.93, 95% CI 0.87–4.28). Planned preterm birth was also associated with postpartum cancers (aOR 1.42, 95% CI 1.05–1.92).

**Table 3:** Outcomes of women with cancer diagnosed during pregnancy compared to women without pregnancy-associated cancer, among 1 308 211 maternities, New South Wales, 1994–2008

Pregnancy outcome	Cancer *N* = 495 *n* (%)	No cancer *N* = 1 307 716 *n* (%)	Crude OR (95% CI)	Adjusted OR (95% CI)
**Place of birth**				
Tertiary hospital	236 (47.7)	516 399 (39.5)	1.41 (1.21, 1.65)	1.43 (1.23, 1.67)
Private hospital	121 (24.4)	272 860 (20.9)	Reference	Reference
Public hospital	138 (27.9)	518 455 (39.6)		
Missing	0 (0.0)	2 (0.0)	–	–
**Induction of labour**				
Yes	141 (28.5)	310 017 (23.7)	1.26 (1.03, 1.53)	1.27 (1.03, 1.56)
No	354 (71.5)	997 319 (76.3)	Reference	Reference
Missing	0 (0.0)	380 (0.0)	–	–
**Mode of delivery**				
Spontaneous vaginal birth	253 (51.1)	864 769 (66.1)	Reference	Reference
Instrumental birth	44 (8.9)	137 415 (10.5)		
Caesarean section	198 (40.0)	304 741 (23.3)	1.89 (1.62, 2.20)	2.08 (1.70, 2.54)
Prelabour	138 (27.9)	170 648 (13.0)	–	–
Intrapartum	60 (12.1)	134 093 (10.3)	–	–
Not stated	0 (0.0)	791 (0.1)	–	–
Duration of pregnancy*				
Any preterm (<37 weeks of gestation)	122 (24.0)	93 045 93 009 (7.0)	–	–
Spontaneous	22 (4.3)	57 309 (4.3)	1.08 (0.67, 1.75)	1.05 (0.65, 1.72)
Planned	100 (19.7)	35 700 (2.7)	10.09 (8.09, 12.58)	11.53 (8.81, 15.11)
≥37 weeks of gestation	385 (76.0)	1 234 465 (93.0)	Reference	Reference
Perinatal death*				
Yes	5 (1.0)	12 019 (0.9)	1.06 (0.40, 2.80)	1.02 (0.40, 2.63)
No	502 (99.0)	1 314 537 (99.0)	Reference	Reference
Missing	0 (0.0)	918 (0.1)	–	–
Birthweight percentiles*				
<10th	54 (10.7)	133 674 (10.1)	1.15 (0.86, 1.54)	1.20 (0.89, 1.61)
10th–90th	373 (73.6)	1 043 541 (78.6)	Reference	Reference
>90th	75 (14.8)	136 415 (10.3)	1.53 (1.18, 1.97)	1.47 (1.14, 1.89)
Missing	5 (1.0)	13 844 (1.0)	–	–

Adjusted for maternal age, socio-economic status, plurality, parity, diabetes and hypertensive disorder. Preterm birth was further adjusted for previous preterm birth. Two women with two cancers diagnosed during pregnancy.

* Based on number of neonates: 507 for which cancer was diagnosed during pregnancy; 1 327 474 for which no cancer was diagnosed.

From 2001 to 2008, 667 019 (99.2%) birth records had a linked hospital record. For women with cancer diagnosed during pregnancy, there were large increases in pregnancy risk of thromboembolic events, sepsis and severe maternal morbidity, but no significant increase in pregnancy risk for obstetric haemorrhage (Table 4). For women with cancer diagnosed after pregnancy, the only significant increase in risk was for postpartum sepsis (aOR 3.33, 95% CI 2.31–4.80).

**Table 4:** Outcomes of women with cancer diagnosed during pregnancy compared to women without pregnancy-associated cancer, among 679 034 maternities, New South Wales, 2001–2008

Pregnancy outcome	Cancer *N* = 287 *n* (%)	No cancer *N* = 678 747 *n* (%)	Crude OR (95% CI)	Adjusted OR (95% CI)
**Obstetric haemorrhage**				
Yes	28 (9.8)	60 022 (8.8)	1.10 (0.74, 1.62)	1.10 (0.74, 1.63)
No	259 (90.2)	618 725 (91.2)	Reference	Reference
**Thromboembolic events**				
Yes	4 (1.4)	878 (0.1)	10.97 (4.10, 29.34)	10.20 (3.81, 27.33)
No	283 (98.6)	677 869 (99.9)	Reference	Reference
**Sepsis**				
Yes	16 (5.6)	9391 (1.4)	4.17 (2.50, 6.94)	4.28 (2.57, 7.13)
No	271 (94.4)	669 356 (98.6)	Reference	Reference
**Severe maternal morbidity**				
Yes	29 (10.1)	10 672 (1.6)	7.05 (4.81, 10.34)	6.89 (4.66, 10.19)
No	258 (89.9)	668 075 (98.4)	Reference	Reference

Adjusted for maternal age, socio-economic status, plurality, parity, diabetes and hypertensive disorder. Two women with two cancers diagnosed during pregnancy.

## Discussion

Consistent with expectation,^6^,^9^,^13^ the incidence of pregnancy-associated cancer increased from 1994 to 2007. Although maternal age was a strong risk factor for cancer, increasing maternal age explained only 14% of the increase. Improved diagnostic techniques, detection and increased interaction with health services during pregnancy might also contribute to higher incidence rates. The cancer incidence was higher than expected among women of reproductive age, and over two-thirds of pregnancy-associated cancers were diagnosed in the 12 months after delivery. The genetic and environmental orgins of pregnancy-associated cancers are likely to pre-date the pregnancy; however, the hormones and growth factors necessary for fetal growth may accelerate tumour growth.

Between 1994 and 2008, the overall incidence of pregnancy-associated cancer in NSW was 137.3 per 100 000 maternities, which is higher than the 100 per 100 000 generally reported in the literature.^1^–^5^ Our reported estimates for the most common types of cancer are also higher than the published international estimates: melanoma of skin (45.7 versus 8.7 per 100 000), breast cancer (28.8 versus 19.3 per 100 000) and thyroid cancer (17.4 versus 14.4 per 100 000), except for cervical (8.4 versus 12.0 per 100 000) and ovarian cancers (3.6 versus 5.2 per 100 000).^1^ Our overall higher incidence partly arises from the predominance of melanoma, for which Australia has the highest incidence in the world.^31^ Slightly higher rates of the other cancers probably reflect our older and recent study population.

The observation that the majority of cancers (72.2%) were diagnosed in the postpartum period has been noted elsewhere.^1^,^9^ Some of these cancers may have been suspected given the high rate of planned preterm delivery for women whose cancer was diagnosed postpartum, but this hypothesis could not be verified in the current data set. Another possible explanation is that the physiologic changes of pregnancy may make cancer more challenging to diagnose, leading to a delay in diagnosis. The distribution of the timing of initial diagnosis is consistent with the contention that postpartum cancers are part of the cancer-in-pregnancy continuum, and are appropriate to be included in the overall incidence.^1^ The steady increase in the number of cancer diagnoses as the duration of pregnancy advanced, which peaked in the 2 months postpartum, may be opportunistic because of pregnancy surveillance.^1^

The increased rate of cancer associated with pregnancy above that expected (based on the rates in the general female population) may be explained in two-fold. First, antenatal and postnatal care, involving a standard protocol of history, physical examination, cervical cytology and blood pressure monitoring, is available for Australian women during and after their pregnancy. On the basis of these routine care visits a screening effect might be expected, thereby increasing the chance of detection of cancer in association with pregnancy. That the melanoma rate is over two times higher than expected in the general population is consistent with such an effect. Second, pregnancy is a proangiogenic state, and it is plausible that the angiogenesis (driven by factors such as placental growth factor and vascular endothelial growth factor) required for successful placentation and pregnancy outcomes contributes to tumerogenesis or growth. *In vitro* placental growth factor increases the proliferation of melanoma cells,^32^ and animal models demonstrate that metastasis is associated with the increased expression of vascular endothelial growth factor receptor.^33^ Furthermore, women with cancer diagnosed during pregnancy were more likely to have multiple pregnancies, and to have large-for-gestational-age infants, even after adjustment for pre-existing or gestational diabetes. High birthweight is an established risk factor for childhood cancers (Wilms’ tumour, infant and childhood leukaemia, osteosarcoma and astrocytoma),^34^ and twins have increased risks of endocrine, bone and breast cancers.^35^,^36^ The postulated mechanism is elevated levels of maternal hormone factors during pregnancy, such as estrogens and insulin-like growth factor I levels, or the aforementioned angiogenic factors.^37^ This mechanism could also predispose maternal cancer, and deserves further investigation. There are few studies that have examined these associations for mothers. We found only one study that examined infant size and breast cancer during pregnancy, which like us, found no increased risk in infant size for breast cancer.^37^ Furthermore, although studies of maternal cancer later in life have examined the risk for women who had multiple pregnancies, the findings have been contradictory, and none have examined pregnancy-associated cancers.^38^

The timing of cancer diagnosis had an effect on adverse pregnancy outcomes. The risks of thromboembolic events, sepsis and severe morbidity, which are recognised cancer complications, were higher among women with cancer diagnosed during pregnancy. Among postpartum cancers, we only demonstrated a significant risk of postpartum sepsis. This is consistent with the fact that these women are more prone to infections and malignancy-related immune suppression, or may have had cancer treatments prior to delivery. The higher rate of caesarean delivery is likely to reflect the standard management plan of certain types of cancer (e.g. cervical cancer) in pregnant women.^9^ Higher rates of planned preterm delivery, to allow the postpartum initiation of cancer treatment, has previously been reported to be common for women with cancer in pregnancy.^9^ The timing of obstetric delivery is a controversial issue surrounding the management of cancer associated with pregnancy. It has been reported that a deliberate delay in treatment is not associated with poor survivorship for pregnant women with early-stage cancer,^5^ and therefore early elective delivery should be carefully considered to ensure the best outcomes for both the mother and the neonate. However, this general finding needs to be assessed by cancer type.

Our findings of higher risks of caesarean delivery and premature birth are consistent with the findings of the population-based Californian studies of pregnancy-associated cancer.^9^ Similar results have also been reported from cancer registry studies on breast, cervical and colorectal cancers, independently.^7^,^10^,^11^ These studies had methodological limitations, however. The Californian studies have assessed pregnancy outcomes solely based on hospitalisation records, in which gestational age and perinatal death are poorly ascertained. Consequently, size at birth was defined by birthweight, which does not differentiate size and maturity. Other limitations included restriction to a specific cancer type, resulting in small sample sizes, and no adjustment for socio-economic status and maternal clinical conditions (hypertensive disorders and diabetes) in assessing pregnancy outcomes.

The strength of our study is the population-based incidence and outcomes of cancer associated with pregnancy derived from large, validated and contemporary data sources. Importantly, there is a complete registration of cancers and births in statutory data collections in NSW. The linkage to cancer registry dating back to 1972 provided the opportunity to assess history of cancer as a risk factor for pregnancy-associated cancer. However, several limitations of our study warrant consideration. First, as early pregnancy loss (miscarriage or abortion) was not registered in the birth data, the number of pregnancy-associated cancers will be somewhat underestimated, and the average gestational age at diagnosis will be over-estimated. Second, we could not examine cancer treatment, as chemotherapy and radiotherapy are primarily provided in outpatient clinics, and are under-ascertained in the hospitalisation records.^39^ Third, history of smoking, alcohol consumption and maternal obesity were not available to provide adjustment for the potential confounding of pregnancy outcomes. Finally, the exact date of cancer diagnosis was not available (month and year only), so the uncertainty between the timing of diagnosis and birth or subsequent adverse outcomes may be as much as 4 weeks.

## Conclusion

In summary, we found a recent increase in pregnancy-associated cancers, such that by 2007 for every 10 000 maternities in Australia 19 women will have an associated cancer diagnosis. Our study provides contemporary cancer incidence rates by cancer type, and informs women and clinicians about the pregnancy outcomes of cancer in pregnancy.

### Disclosure of interests

The authors declare no conflicts of interest.

### Contribution to authorship

CLR and ES conceived the study. All authors participated in the study design, planning of analysis and interpretation of the results. YYL was involved in data preparation, statistical analyses and drafting the article. TD provided statistical expertise and a critical review of the article. CLR provided clinical expertise, helped to draft the article and coordinated the study. JM, JY, ES and KB provided clinical expertise and a critical review of the article. All authors read and approved the final article.

### Details of ethics approval

The study was approved by the NSW Population and Health Services Research Ethics Committee on 6 October 2009 (ref. no. 2009/08/172).

### Funding

The study was supported by the Australian Government National Collaborative Research Infrastructure Strategy’s Population Health Research Network. Christine Roberts is supported by an National Health and Medical Research Council Senior Research Fellowship.
